# Exploring the Connections between Medical Rehabilitation, Faith and Spirituality

**DOI:** 10.3390/healthcare12121202

**Published:** 2024-06-15

**Authors:** Laszlo Irsay, Viorela Mihaela Ciortea, Theodor Popa, Madalina Gabriela Iliescu, Alina Deniza Ciubean

**Affiliations:** 1 Department of Clinical Rehabilitation, “Iuliu Hatieganu” University of Medicine and Pharmacy, 400347 Cluj-Napoca, Romania; laszlo.irsay@umfcluj.ro (L.I.); alina.ciubean@umfcluj.ro (A.D.C.); 2 Department of Rehabilitation, Clinical Rehabilitation Hospital, 400347 Cluj-Napoca, Romania; 3 Techirghiol Sanatorium Rehabilitation Department, Faculty of Medicine, Ovidius University of Constanta, 906100 Constanta, Romania; madalina.iliescu@365.univ-ovidius.ro

**Keywords:** medical rehabilitation, faith, spirituality, healthcare professionals, ethics

## Abstract

(1) Background: Patients who undergo a medical rehabilitation treatment are often facing a physical, emotional and spiritual crisis, mostly due to pain, loss of limb functionality, the memory of the pre-disease days or questions about their role and value in life. Most of the time, the physician does not have the ability to deal with these issues or to provide the expected responses. The aim of this study was to analyze the patient’s perception on spirituality and faith while going through a medical rehabilitation program. (2) Methods: The current study included 173 patients treated in the Rehabilitation Department of the Clinical Rehabilitation Hospital in Cluj-Napoca, Romania. Of them, 91 comprised the study group and were assessed in 2023, while 82 comprised the control group and were assessed in 2007. All patients answered a 34-item questionnaire designed by the authors regarding the role of religion, spirituality and prayer in their post-disease life. (3) Results: The results show that 99% of the patients assessed believe in God, 80% pray every day, 50% have less pain after praying and 44% trust their priest the same as they trust their doctor. When comparing groups, results from 2023 show that more patients pray every day, while fewer are afraid of dying, think their disease is serious or wish for the medical team to pray with them, compared to 2007. (4) Conclusions: The physician should not neglect the faith of the patient and should use it to achieve a better rehabilitation outcome.

## 1. Introduction

Over the past centuries, prayer and meditation have been used to relieve human suffering. Until recently, spirituality and medicine had a tight connection a long period of time. In Hindu American civilizations, the healers were also spiritual leaders. In Chinese and Ayurveda traditional medicines, evaluation of spirit and soul is considered an important aspect of treatment. Since the Darwinism era, science and spirituality have had a less constructive interrelation [1]. In the Roman days, the gods were the ones who contributed significantly to both the well-being and destruction of an individual, based on their degree of satisfaction of their requests. In the witches era, people with body deformities or strange diseases were considered cursed or partners with the devil. Even in the Bible, the “unclean” people (e.g., lepra) were considered cursed for not keeping a tighter connection with God. Looking at history, it is safe to assume that religion has had a significant impact on the development of humanity [1].

Patients who undergo a medical rehabilitation treatment are often facing a physical, emotional and spiritual crisis. The presence of pain, either chronic or acute, losing a limb or a function of a body segment, the memory of the pre-disease days, and questions about their role and value in life are just a few of the problems that these patients deal with. Most of the time, the physician is not equipped with the ability to deal with these types of issues or to provide the expected responses. Spiritual matters tend to surface indirectly and informally during interactions with patients due to perceived barriers such as privacy concerns, professional boundaries, and staff discomfort, largely stemming from the conflation of spirituality with religion. Health professionals suggest enhancing spirituality’s role by providing physical spaces for addressing spiritual needs and incorporating it into rehabilitation processes like assessments and goal planning. Similar challenges are observed in broader healthcare contexts, where despite recognizing the importance of spirituality, many health professionals lack confidence in addressing it, citing barriers like time constraints, confusion between spirituality and religion, institutional barriers, and insufficient knowledge or skills [2,3,4,5].

Defining the notions of praying, meditation and spirituality can be a difficult task. Webster defines prayer as “a close approach to a divinity through words or by thought”. The current literature describes different types of prayer: confession, request, complaint, begging, worship and thanking. Meditation seems to be the other side of the coin, as it is a technique of focusing ones attention on an object, sound, thought, prayer, movement or breath. The main objective seems to be the minimalization of the external stimuli and focus on internal ones. Praying is mainly perceived as a way of communicating with divinity, while meditation is actually listening to it. Either way, there is a superposition between the two [6].

It is now a known fact that religion offers support during the medical rehabilitation process. Religious coping is defined as the use of behavioral and cognitive techniques in stressful life events in a multidimensional construct with positive and negative effects on outcomes, while religiosity is considered a use of individual beliefs, values, practices and rituals related to faith. There are multiple studies that prove the efficiency of religious coping in different areas of medical rehabilitation, like in pulmonary [7], depression [8], addiction [9], cardiovascular [10], chronic kidney disease [11,12], epilepsy [13] and chronic pain [14]. Recent research conducted by Jones et al. (2018) highlights that while healthcare professionals acknowledge the significance of spirituality in aiding the adjustment of clients and their families after spinal cord injury, it is often not integrated into rehabilitation practices [15]

Therefore, the purpose of the present study was to assess how spirituality and faith are perceived by patients undergoing a rehabilitation program, their vision on praying and its role during the process.

## 2. Materials and Methods

The current study is observational, case–control type, and included a total of 173 patients, both female and male, who were treated as inpatients at the Rehabilitation Unit of the Clinical Rehabilitation Hospital in Cluj-Napoca, Romania. Of them, 82 patients were assessed in 2007 and 91 patients in 2023. All patients underwent a 10–15-day rehabilitation program.

The inclusion criteria consisted of (1) pain for more than 6 months, regardless of etiology and/or (2) the presence of a motor deficit that alters the capacity of self-care and performance of activities of daily living; (3) the ability to answer questionnaires (lack of dementia or other major cognitive impairments).

The patients included in this study were asked to complete a 34-item questionnaire designed by the authors, with *Yes*, *No* or *I don’t know* answers. Clinical data were also collected, like age, gender, geographic area, school stage and religion. The questionnaire was completed by the patient while alone, and clinical data were collected by the treating physician.

Data for the control group were collected in 2007, and data for the current study group were collected in 2023.

Patient data were entered in the Microsoft Office Excel 2010 program and statistical analysis was performed using Graph Pad Prism 8. Quantitative data were tested for normality of distribution and expressed as mean ± standard deviation. Qualitative data were expressed as frequency and percent. Comparison between groups was performed using the Student *t*-test. A *p*-value < 0.05 was considered statistically significant.

All subjects were informed of the characteristics of this study and were required to sign an informed consent document approved by the Ethics Committee of the “Iuliu Hatieganu” University of Medicine and Pharmacy. The questionnaires ensured anonymity. All subjects participated voluntarily in this study.

## 3. Results

A total of 173 patients met the inclusion criteria and were included in this study. Of them, 91 comprised the study group, while 82 comprised the control group. In the study group, 76% were female, the mean age was 61.5 years and 77.64% were Orthodox. In the control group, 58.5% were female, the mean age was 53.2 years and 73.17% were Orthodox. The main characteristics of the patients included in this study are shown in Table 1.

Results from analysis of the answers of the questionnaire from the study group show that 99% of the patients assessed believe in God, 80% pray every day, 50% have less pain after praying, 69% believe that only God can heal them and 44% trust their priest the same as they trust their doctor.

When comparing results between the groups, as shown in Table 2, it was observed that in the study group, fewer patients think that their disease is a curse or that their disease is serious (*p* = 0.008 and *p* = 0.003, respectively). When asked about whether they wished for the medical team (nurses, doctor) to pray with them, fewer patients answered in a positive manner than in 2007 (*p* = 0.008). In the study group, 80% of the patients answered positively to praying every day, compared to 67.1% in 2007 (*p* = 0.043), while less admitted to trusting their priest as they trust their doctor (*p* = 0.021). Also, in the study group, fewer patients admitted of being afraid of dying, while more patients said that they carry a saint image/cross or that they do not go to church because church is wherever they pray (all *p* < 0.05). In the other questions, no statistical significance was found between groups.

## 4. Discussion

Religion is the path by which life provides meaning and significance in human existence. It was previously shown that there are multiple common elements across different religions: belief in an intelligent supernatural being, a global interpretation of life, belief in the existence of an afterlife, the presence of a moral code that when not respected leads to sanctions by the supernatural being, praying, rituals, sacred objects and places, fear connected to religious feelings, mystical experiences and revelations [16].

If meditation and prayer can somehow be defined, spirituality is a more complex concept. It can be defined as a connection to a universal force, which can be God, a divinity, nature, the universe, all that have a higher power than any other force and play a guidance role. One definition of spirituality refers to the non-body and non-mental dimension of a person, which is the source of unity and understanding [17]. Another definition, more functional, is that spirituality is the continuous unity of mind, body and spirit that shapes the existence of each person. We perceive temperature, pression, proprioception and pain through the body unit. Mentally, we perceive satisfaction, fear and anxiety. Joy, love and fulfillment are considered part of the spiritual unit. Even though none of these originate from the physical or mental unit, they are filtered at this level and have a significant impact on the functionality of the physical and mental components [18].

On the spiritual level, negative sides are also included, like negative intuition and the belief that “something is wrong” or that pain has no physical correspondent. From a rehabilitation point of view, spirituality is perceived as an interior resource of force, courage, hope, joy and good physical tonus. In medicine, approaching spirituality can be associated with discomfort because of the differences in various religions, the fear of approaching an intimate topic and the apparent lack of connection between science and spirit [8,9].

There is a growing body of research suggesting that spirituality and faith can significantly impact health outcomes. Studies have shown that individuals who engage in spiritual practices or hold strong religious beliefs often exhibit better coping mechanisms, lower levels of stress and improved overall well-being. In the context of medical rehabilitation, these factors can contribute to a more positive recovery experience and better treatment outcomes. By integrating spiritual care into rehabilitation programs, healthcare providers can support patients in drawing upon their spiritual resources to cope with the challenges of illness or disability, fostering a sense of hope, meaning and resilience throughout the recovery process [19,20].

There are multiple studies that investigate this topic, but few within the medical rehabilitation domain. Enquist et al. (1997) performed a study on occupational therapists and showed that 84% of them believe that spirituality is an important side of life, but most of them feel that it does not fit with their area of expertise [21]. Another study, on a wide cohort of 1221 individuals from the rehabilitation domain (physicians, physical therapists) indicated that 56% believed that praying has a role on the clinical side, but only half of them really used it [22]. Most studies concluded that patients wish they had a tighter doctor–patient spiritual relation and express a desire to discuss spirituality and view it as part of the health professional’s role. Addressing these issues could involve staff training to improve understanding and confidence, as demonstrated in palliative care settings. Another approach could be implementing structured spiritual needs assessments, although careful collaboration and training with staff would be necessary due to their reluctance [21,22,23,24]. This recognition is based on research that shows significant correlations between spirituality and positive health outcomes such as increased quality of life, resilience, mental well-being and life satisfaction. Despite this understanding, many healthcare professionals feel unprepared or hesitant to address their patients’ spiritual needs in their daily practice. The primary reasons for this reluctance are a lack of spiritual care training and a lack of available time [25,26].

Spirituality has been defined in various ways in the healthcare context, with varying degrees of emphasis on its relationship to religious beliefs. Spirituality was defined based on the consensus reached at a conference of palliative care experts, who described it as “the aspect of humanity concerned with the search for meaning and purpose, and the experience of interconnectedness with the present moment, oneself, others, nature, and the sacred or significant”. This broad definition acknowledges that people can derive meaning and connection from a variety of sources, including religious faith, the natural world, interpersonal relationships, music, art and self-reflection. Spiritual care, similarly broadly defined, is characterized as person-centered care aimed at helping individuals rediscover hope, resilience and inner strength during times of illness, injury, transition and loss [27,28,29].

But most importantly, the studies that aim to analyze the efficiency of prayer tend to confront certain particular difficulties and biases. In the matter of a case–control study, in which the cases pray, it is unsure whether or not the controls do the same. Also, “quantity and quality” are aspects for which it is unclear as to whether quantification is needed or even relevant in the context of such a study. Most studies deal with these questions. Praying is mostly used as a way of coping to manage stress and other factors associated with disease and treatment [30].

Another study revealed the fact that prayer was used the most often as a self-care form of treatment in patients with osteoarthritis [31]. Researchers often tend to underline the fact that religion, spirituality and faith can be as important as eating habits, exercise and stress management for achievement of a good health status [32,33].

There are sufficient data to show that even in severe conditions, like patients with spinal cord injury, who have severe disability, spirituality was associated with improved quality of life, life satisfaction, mental health and resilience [34]. In other pathologies, like cardiac rehabilitation, the correlation was more empirical, but with an important impact on the psychosocial and emotional status of the patients [35].

In a study conducted in the USA in 1999 that included 1000 adults, 79% of the participants considered faith as an important factor to achieve healing and 63% believed that it is the duty of doctors to talk with patients about faith. As for the desire of the doctor to pray alongside them, 48% of the patients answered in a positive manner [36].

At a United States family physician congress in 1996, of the 296 doctors who were interviewed, 99% considered religious belief as an important healing force and 75% thought that praying for a patient could promote his/her rehabilitation. Moreover, there are doctors who believe that the so-called wall that separates medicine and religion will fall apart and think that the “future medicine will consist of prayer and Prozac” [37,38].

In a publication by the American Medical Association, the authors believed that with each consult, the doctor should ask the patient what can be done to support his/her faith. Most rehabilitation physicians admit that during their training, no aspect of dealing with the patient from a spiritual point of view is taught, suggesting that a different profession could be more appropriate in dealing with these patient needs [7].

How religion works and acts remains a mystery. A variety of studies conducted on priests and monks (both male and female), where the researchers assumed a high religious devotion, showed that morbidity and mortality was lower than observed in the general population. But a limitation of these studies is that the degree to which religion and faith intervene is not known, nor whether other factors have an impact, e.g., lack of smoking and alcohol consumption, stress, meat consumption in some cases and sexual behavior [39,40,41,42].

Being religious led to a faster recovery in female patients with hip fracture, mostly for gait restoration, but it is important to mention that in the study, the age of the patients was not considered a factor, which could be of critical significance, from a rehabilitation point of view [43]. Also, the results of the study are contradictory, due to the fact that certain religious beliefs prohibit meat consumption, which leads to normal cholesterol levels, as other researchers found hypertriglyceridemia in 17% of the cases of the same religion [44].

In many studies, comparisons between faithful and not faithful patients do not achieve sufficient statistical significance. The typical example is a study conducted in an emergency room setting for coronary heart disease. Lack of a unanimous definition of religiousity or spiritual activity is a major problem in conducting and comparing these studies [45,46].

Another important problem comes from an ethical point of view. Does the doctor have the right to influence or ask questions regarding a patient’s religious beliefs? On the other side, if this communication could lead to a better understanding of patients’ problems, and could lead to a faster and complete recovery, this theme should be approached. The spiritual needs of patients should be taken into consideration for better outcomes, a better quality of life and to satisfy the patient. There is however the risk of harming the patient by suggesting that a lack of faith could have led to the disease or that the disease is the result of moral failures. Many doctors think that approaching the patient from a spiritual point of view is not part of their profession, but this theme should be discussed when the patient desires [47,48,49,50].

The mechanism by which prayer works is not known. Expert meditators have shown significant homeostasis alterations: lower heart and respiratory rate, lower oxygen consumption, lower lactate levels, EEG modifications and modifications of skin resistance to galvanic current. All these were interpreted as responses to relaxation. Recently, functional magnetic resonance showed a global decrease in signaling. Specifically, meditation activates the neural structures responsible for attention and those involved in autonomic response [1]. A systematic review conducted by Simao et al. (2016) indicates that incorporating prayer into clinical practice can yield various positive effects, such as reduced anxiety and depression, improved physical functioning, lower mortality rates in patients with bloodstream infections, and shorter stays in the coronary care unit for cardiac patients [51]. Neuropsychoimmunology, a field exploring the intricate interactions between the nervous, psychological and immune systems, has increasingly drawn attention to the potential impacts of prayer and faith on health. Research suggests that religious practices, such as prayer, can influence neurobiological pathways, thereby affecting stress responses and immune function. For instance, prayer and meditation have been associated with reduced levels of cortisol, a stress hormone, and enhanced immune response, possibly due to their role in promoting relaxation and reducing anxiety [52]. Moreover, faith and spirituality are believed to provide psychological benefits, such as a sense of purpose and community, which can buffer against stress and improve overall well-being [53]. Studies have also shown that individuals engaging in regular prayer or meditation exhibit increased activity in brain regions associated with attention and emotional regulation, such as the prefrontal cortex [54,55]. These findings highlight the complex interplay between psychological states induced by prayer and faith and their potential to modulate immune function, underscoring the importance of integrating neuropsychoimmunological perspectives in understanding the health benefits of religious practices.

The modifications can be somehow quantified. More interesting and mysterious is how praying can influence ones health status even when a person does not know what disease he/she is praying for. One hypothesis is that when the person prays, the immune and endocrine systems are stimulated. But all these are only speculations and there is a chance that the action mechanism is an unknown scientific pathway [36].

Robert Gerber, the father of energetic medicine, who describes the physiological alterations as consequences of energetic variations, uses Einstein’s equation (E = m∙c^2^), knowing that energy and matter are interconvertible. They explain why prayer (a form of energy) can change certain structures, so it can intervene on a physical level [37].

Even in the strongest studies conducted so far, correlation between religion, spirituality and health is mainly weak and inconstant. We consider it premature to promote faith and religion as a complementary treatment. Still, between the extreme attitude of rejecting any idea that religion and faith could bring a certain degree of comfort in some patients who deal with a certain disease, and the attitude that recommends patients pursue a religious activity, exists an intermediate attitude.

Particularly, the rehabilitation process is a personal choice; one role of the physician is to identify the needs of the patient, and his/her faith is very important. In this case, the aim is for the rehabilitation program to succeed, not for the patient to have faith in some form of divinity. Some practical rehabilitation guidelines include certain important steps: the patient and the doctor should establish the rehabilitation steps together, the main plan should be focused on discouraging any dependence level, while spirituality and cultural differences should be taken into consideration, as the physician aims to gain the patient’s trust.

The current study design exhibits several significant limitations that could affect the validity and reliability of the findings. Firstly, the substantial 15-year gap between the control and test subjects introduces considerable variability in patient behavior, belief systems, the extent of faith, and the frequency and regularity of prayer. This temporal disparity means that societal and cultural changes over the years have likely influenced these factors, yet they have not been investigated or quantified. Additionally, the lack of a standardized questionnaire poses a serious methodological issue. Variations in question phrasing, response options and the overall structure of the questionnaire can lead to inconsistencies in the data collected. Even if the same person administered the questionnaire, the differences in how it is administered over such a long period could result in variations in responses due to changes in the administrator’s approach, interpretation or emphasis. Furthermore, the absence of a qualitative approach leaves out rich, detailed data that could provide deeper insight into patient experiences and beliefs, which are crucial for understanding the context of prayer’s impact. Other biases, such as recall bias, where participants may not accurately remember past events or experiences, and selection bias, where the sample may not represent the broader population, could not be addressed adequately. To improve subsequent studies, a more consistent and contemporaneous comparison group should be used to eliminate the temporal gap. Implementing a standardized, validated questionnaire would enhance reliability and comparability. Incorporating qualitative methods, such as interviews or focus groups, could provide a more comprehensive understanding of the patients’ perspectives and the nuances of their prayer practices. Additionally, employing mixed-method approaches and triangulating data from various sources could help mitigate biases and provide a more robust and holistic view of the phenomena under investigation.

As Robert Gerber states: “A medical system that denies or ignores the existence of spirituality will be incomplete, as it leaves on the outside one of the most important factors of human existence”.

## 5. Conclusions

The spirituality and faith of patients undergoing a medical rehabilitation program are and will remain an interior force hard to assess and quantify. They cannot be neglected and should be considered as a potential resource to achieve better outcome during medical rehabilitation.

## Figures and Tables

**Table 1:** Clinical characteristics of the patients included in this study.

Clinical Characteristics		Study Group (*n* = 91)	Control Group (n = 82)
Gender (%)	Female	76	58.5
	Male	24	41.5
Age (years) mean ± SD		61.5 ± 11.6	53.8 ± 14.2
Geographic area (%)	Urban	53	44
	Rural	47	56
School stages (%)	Middle	16.8	26.8
	High	4.2	18.2
	Professional	47	36.5
	Graduate	28.9	18.2
Religion (%)	Orthodox	77.64	73.17
	Other	22.36	26.83

**Table 2:** Comparison of answers between study and control group.

Question	Study Group (2019)			Control Group (2007)			*p*-Value
	Yes	No	I Don’t Know	Yes	No	I Don’t Know	
My disease is a curse (%)	2.2	87.7	10.1	3.7	67	29.3	0.008
I think my disease is serious (%)	19.3	61.4	19.3	46.3	37.8	15.9	0.003
I wish the medical team (nurses, doctor) prayed with me (%)	25.3	58.6	16.1	48.8	39	12.2	0.008
I am afraid of dying (%)	18.1	73.9	8	31.7	64.6	3.7	0.025
I pray every day (%)	80	18.9	1.1	67.1	29.3	3.7	0.043
I trust the priest the same as I trust my doctor (%)	40.9	44.3	14.8	57.3	35.4	7.3	0.021
I always have a saint image/cross with me (%)	64	32.6	3.4	42.7	54.9	2.4	0.016
I do not go to church because church is wherever I pray (%)	44.3	50.8	4.9	34.9	46	19.1	0.049

## Data Availability

The original contributions presented in this study are included in the article; further inquiries can be directed to the corresponding authors.

## References

[1] Cotter C.A., Stanley F.W., Avital Fast (2003). Spangenberg Valerie, Mulford Gregory: Prayer, Meditation, and Spirituality in Rehabilitation. Alternative Medicine and Rehabilitation.

[2] Oxhandler H., Parrish D., Torres L., Achenbaum W. (2015). The integration of clients’ religion and spirituality in social work practice: A national survey. Soc. Work.

[3] Best M., Butow P., Olver I. (2016). Why do we find it so hard to discuss spirituality? A qualitative exploration of attitudinal barriers. J. Clin. Med..

[4] Hilbers J., Haynes A.S., Kivikko J.G. (2010). Spirituality and health: An exploratory study of hospital patients’ perspectives. Aust. Health Rev..

[5] Austin P., Macleod R., Siddall P.J., McSherry W., Egan R. (2016). The ability of hospital staff to recognise and meet patients’ spiritual needs: A pilot study. J. Study Spiritual..

[6] (1993). Merriam-Webster’s Collegiate Dictionary.

[7] da Silva G.P., Nascimento F.A., Macêdo T.P., Morano M.T., Mesquita R., Pereira E.D. (2018). Religious coping and religiosity in patients with COPD following pulmonary rehabilitation. Int. J. Chron. Obstruct. Pulmon. Dis..

[8] Stearns M., Nadorff D.K., Lantz E.D., McKay I.T. (2018). Religiosity and depressive symptoms in older adults compared to younger adults: Moderation by age. J. Affect. Disord..

[9] Beraldo L., Gil F., Ventriglio A., de Andrade A.G., da Silva A.G., Torales J., Gonçalves P.D., Bhugra D., Castaldelli-Maia J.M. (2019). Spirituality, Religiosity and Addiction Recovery: Current Perspectives. Curr. Drug Res. Rev..

[10] Trevino K.M., McConnell T.R. (2015). Religiosity and Spirituality During Cardiac Rehabilitation: A longitudinal evaluation of patient-reported outcomes and exercise capacity. J. Cardiopulm. Rehabil. Prev..

[11] Bragazzi N.L., Puente G.D. (2013). Chronic Kidney Disease, Spirituality and Religiosity: A Systematic Overview with the List of Eligible Studies. Health Psychol. Res..

[12] Gerogianni G., Babatsikou F., Polikandrioti M., Grapsa E. (2019). Management of anxiety and depression in haemodialysis patients: The role of non-pharmacological methods. Int. Urol. Nephrol..

[13] Lin C.Y., Saffari M., Koenig H.G., Pakpour A.H. (2018). Effects of religiosity and religious coping on medication adherence and quality of life among people with epilepsy. Epilepsy Behav..

[14] Ferreira-Valente A., Sharma S., Torres S., Smothers Z., Pais-Ribeiro J., Abbott J.H., Jensen M.P. (2019). Does Religiosity/Spirituality Play a Role in Function, Pain-Related Beliefs, and Coping in Patients with Chronic Pain? A Systematic Review. J. Relig. Health.

[15] Jones K.F., Dorsett P., Briggs L., Simpson G.K. (2018). The role of spirituality in spinal cord injury (SCI) rehabilitation: Exploring health professional perspectives. Spinal Cord Ser. Cases.

[16] Edwards J., Goen C.C. (1972). The Great Awakening.

[17] Hiatt J.F. (1986). Spirituality, medicine, and healing. South. Med. J..

[18] Gagnon D. (2003). A Spiritual Psychosocial Biological Model of Rehabilitation: The Importance of Existential Meaning.

[19] Sulmasy D.P. (2002). A biopsychosocial-spiritual model for the care of patients at the end of life. Gerontologist.

[20] Jones K.F., Dorsett P., Simpson G., Briggs L. (2018). Moving forward on the journey: Spirituality and family resilience after spinal cord injury. Rehabil. Psychol..

[21] Engquist D.E., ShortDeGraff M., Gliner J., Oltjenbruns K. (1997). Occupational therapist beliefs and practices with regard to spirituality and therapy. Am. J. Occup. Ther..

[22] Curlin F.A., Sellergren S.A., Lantos J.D., Chin M.H. (2007). Physicians’ observations and interpretations of the influence of religion and spirituality on health. Arch. Intern. Med..

[23] Schoenberger N.E., Matheis R.J., Shiflett S.C., Cotter A.C. (2002). Opinions and practices of medical rehabilitation professionals regarding prayer and meditation. J. Altern. Complement. Med..

[24] Hodge D.R. (2015). Administering a two-stage spiritual assessment in healthcare settings: A necessary component of ethical and effective care. J. Nurs. Manag..

[25] Jones K.F., Pryor J., Care-Unger C., Simpson G.K. (2020). Rehabilitation health professionals’ perceptions of spirituality and spiritual care: The results of an online survey. NeuroRehabilitation.

[26] Best M., Butow P., Olver I. (2016). Palliative care specialists’ beliefs about spiritual care. Support Care Cancer.

[27] de Brito Sena M.A., Damiano R.F., Lucchetti G., Peres M.F. (2021). Defining spirituality in healthcare: A systematic review and conceptual framework. Front. Psychol..

[28] Davis D.E., Rice K., Hook J.N., Van Tongeren D.R., DeBlaere C., Choe E., Worthington E.L. (2015). Development of the sources of spirituality scale. J. Couns. Psychol..

[29] Puchalski C., Ferrell B., Virani R., Otis-Green S., Baird P., Bull J., Chochinov H., Handzo G., Nelson-Becker H., Prince-Paul M. (2009). Improving the quality of spiritual care as a dimension of palliative care: The report of the consensus conference. J. Palliat. Med..

[30] Saudia T.L., Kinney M.R., Brown K.C. (1991). Health locus of control and helpfulness of prayer. Heart Lung.

[31] Arcury T.A., Bernard S.L., Jordan J.M., Cook H.L. (1996). Gender and ethnic differences in alternative and conventional arthritis remedy use among community-dwelling rural adults with arthritis. Arthritis Care Res..

[32] Koenig H.G., George L.K., Cohen H.J., Hays J.C., Larson D.B., Blazer D.G. (2002). The relationship between religious activities and cigarette smoking in older individuals. J. Gerontol..

[33] Koenig H.G. (1997). Is Religion Good for your Health?.

[34] Jones K., Simpson G.K., Briggs L., Dorsett P. (2016). Does spirituality facilitate adjustment and resilience among individuals and families after SCI?. Disabil. Rehabil..

[35] Nadarajah S., Berger A.M., Thomas S.A. (2013). Current status of spirituality in cardiac rehabilitation programs: A review of literature. J. Cardiopulm. Rehabil. Prev..

[36] King D., Bushwick B. (1994). Beliefs and attitudes of hospital inpatients about faith healing and prayer. J. Fam. Pract..

[37] Matthews D.A., Larson D.B. (1997). Faith and medicine: Reconciling the twin traditions of healing. Mind/Body Med..

[38] Larson D.D., Larson S.S. (1991). Religious commitment and health: Valuing the relationship. Second. Opin. Health Faith Ethics.

[39] Michalek A.M., Mettlin C., Priore R.L. (1981). Prostate cancer mortality among Catholic priests. J. Surg. Oncol..

[40] Timio M., Lippi G., Venanzi S. (1997). Blood pressure trend and cardiovascular events in nuns in a secluded order: A 30-year follow-up study. Blood Press..

[41] Gardner J.W., Lyon J.L. (1982). Cancer in Utah Mormon men by lay priesthood level. Am. J. Epidemiol..

[42] de Gouw H.W.F.M., Westndrop R.G.J., Kunst A.E., Mackenbach J.P., Vandenbrouke J.P. (1996). Decreased mortality among contemplative monks in the Netherlands. Am. J. Epidemiol..

[43] Pressman P., Lyons J.S., Larson D.B., Strain J.J. (1990). Religious belief, depression and ambulation status in elderly women with broken hips. Am. J. Psychiatry.

[44] Walden R.T., Schaefer L.E., Lemon F.R., Sunshine A., Wynder E.L. (1964). Effect of environment on the serum cholesterol-triglyceride distribution among Seventh-day Adventists. Am. J. Med..

[45] Byrd R. (1988). Positive therapeutic effects of intercessory prayer in a coronary care unit population. South Med. J..

[46] Harris W.S., Gowda M., KoIb J.W., Strychacz C.P., Vacek J.L., Jones P.G., Forker A., O'Keefe J.H., McCallister B.D. (1999). A randomized, controlled trial of the effects of remote, intercessory prayer on outcomes in patients admitted to the coronary care unit. Arch. Int. Med..

[47] Gerber R. (2001). Vibrational Medicine.

[48] Marshall C.A., Hoskie R. (1999). Disability and rehabilitation: A context for understanding the American Indian experience. Lancet.

[49] McClain C.S., Rosenfeld B., Breitbart W. (2003). Effect of spiritual well-being on end-of-life despair in terminally-ill cancer patients. Lancet.

[50] Daaleman P.T., Frey B.B., Wallace D. (2002). Spirituality index of well-being scale: Development and testing of a new measure. J. Fam. Pract..

[51] Simão T.P., Caldeira S., De Carvalho E.C. (2016). The effect of prayer on patients’ health: Systematic literature review. Religions.

[52] Koenig H.G. (2012). Religion, spirituality, and health: The research and clinical implications. ISRN Psychiatry.

[53] Ai A.L., Bolling S.F., Peterson C. (2000). The use of prayer by coronary artery bypass patients. Int. J. Psychol. Relig..

[54] Dobrakowski P., Blaszkiewicz M., Skalski S. (2020). Changes in the Electrical Activity of the Brain in the Alpha and Theta Bands during Prayer and Meditation. Int. J. Environ. Res. Public Health.

[55] Maron-Katz A., Ben-Simon E., Sharon H., Gruberger M., Cvetkovic D. (2014). A neuroscientific perspective on meditation. Psychology of Meditation.

